# MP:PD—a data base of internal packing densities, internal packing defects and internal waters of helical membrane proteins

**DOI:** 10.1093/nar/gkt1062

**Published:** 2013-11-03

**Authors:** Alexander Rose, Dominic Theune, Andrean Goede, Peter W. Hildebrand

**Affiliations:** ^1^Charité University Medicine Berlin, Institute of Medical Physics and Biophysics, ProteinFormatics Group, Charitéplatz 1, 10117 Berlin and ^2^Charité University Medicine Berlin, Institute for Physiology, Structural Bioinformatics Group, Lindenberger Weg 80, 13125 Berlin

## Abstract

The membrane protein packing database (*MP:PD*) (http://proteinformatics.charite.de/mppd) is a database of helical membrane proteins featuring internal atomic packing densities, cavities and waters. Membrane proteins are not tightly packed but contain a considerable number of internal cavities that differ in volume, polarity and solvent accessibility as well as in their filling with internal water. Internal cavities are supposed to be regions of high physical compressibility. By serving as mobile hydrogen bonding donors or acceptors, internal waters likely facilitate transition between different functional states. Despite these distinct functional roles, internal cavities of helical membrane proteins are not well characterized, mainly because most internal waters are not resolved by crystal structure analysis. Here we combined various computational biophysical techniques to characterize internal cavities, reassign positions of internal waters and calculate internal packing densities of all available helical membrane protein structures and stored them in *MP:PD*. The database can be searched using keywords and entries can be downloaded. Each entry can be visualized in *Provi*, a *Jmol*-based protein viewer that provides an integrated display of low energy waters alongside membrane planes, internal packing density, hydrophobic cavities and hydrogen bonds.

## INTRODUCTION

Communication between cells and different cell compartments is governed by helical membrane proteins. These proteins are involved in many different cellular processes, such as signal transduction, pumping, channelling, light harvesting, translocation and proteolysis (1). During the past decade, attempts to obtain 3D structures of helical membrane proteins have achieved sustained success. As a consequence, the number and diversity of high-resolution membrane protein structures has increased substantially (2). Still, most membrane proteins are only elucidated at modest resolution so that structural details, such as side chain packing or internal waters are often not adequately resolved. Here we used a combination of various biophysical tools to calculate internal atomic packing densities, characterize internal cavities and reassign positions of internal waters in helical membrane proteins, and stored this information in a database called membrane protein packing database (*MP:PD*).

Statistical analysis of helical membrane protein structures has revealed that membrane proteins contain a considerably large number of water-sized or even larger internal packing defects (‘internal cavities’) (3). As a consequence, helical membrane proteins are not tightly packed (4,5). Depending on their polar or hydrophobic nature, internal cavities of proteins can be filled with internal water molecules, gas or may even be empty (6,7). Internal cavities were found to collapse under high pressure suggesting that they are important structural elements of protein folding and unfolding (8). Conformational sub-states of proteins differ in their relative partial molar volume and isothermal compressibility as revealed by high-pressure EPR (9). Changes in population of protein states or sub-states are therefore likely accompanied by local changes of packing densities or by modifications of internal cavities. This hypothesis is in general agreement with the finding that internal cavities cluster at functionally important protein sites such as hinge regions of channels and transporters or along the pores of channels (3,5). Placement of bulky residues at internal cavities changes the activation profile of G-protein coupled receptors (GPCR) (10) and enhances the thermal stability of a given state (11). These mutational experiments suggest that the suboptimal internal packing of proteins is generally an indispensable structural element of membrane protein function.

By providing alternative hydrogen bonding partners, internal waters are predicted to stabilize transition states of helical membrane proteins in which the hydrogen-bonding network is significantly altered (5,12). In this manner, internal waters likely codetermine the structural reorganisations occurring during activation of GPCRs (12–14). Conformational changes, triggered by the shifting of backbone hydrogen bonding partners at kinks of transmembrane helices (15), may be facilitated by nearby internal water molecules (12). Another specific functional role of internal water molecules is that they can facilitate proton transfer reactions (16–18). The functional role of internal cavities containing one or more water molecules again appears to be different from those containing no water. Residues neighbouring empty or only partially filled internal cavities likely retain a higher conformational flexibility than those located in tightly packed regions of proteins. As a result, the loss of conformational entropy for cavity forming residues should be smaller, partially compensating for the positive enthalpy of forming a void inside a protein. Thus, a comprehensive description of the size, accessibility and polarity of internal cavities is required.

Here we applied the Voronoi cell method to calculate internal atomic packing densities (19) and the MSMS tool to allocate internal cavities and differentiate them from exposed cavities (20). Cavities placed in protein clefts or within channels and pores restricted by narrow entranceways were included. Spherical probes of 1.4 or 1.7 Å were used to calculate the surface of polar cavities or hydrophobic cavities, respectively. Positions of internal waters filling internal cavities were calculated based on their interaction energies with the surrounding atoms using the program DOWSER (21). Internal cavities, newly assigned water molecules and their hydrogen bonding networks can be downloaded or visualized along with other structural information with *Provi*, a *Jmol*-based protein viewer.

## DATABASE CONTENT AND ACCESS

### List of helical membrane proteins

*MP:PD* is a sub-dataset of the RCSB PDB (22) and lists only proteins with at least one transmembrane helix. It is comprised of presently 1546 alpha helical transmembrane proteins derived from the OPM (23), the PDBTM (24) and the MPstruc (http://blanco.biomol.uci.edu/mpstruc/) database. OPM and PDBTM employ different algorithms to detect membrane proteins in the RCSB PDB, while MPstruc is curated manually. The OPM database includes transmembrane protein complexes and selected monotopic, peripheral membrane proteins and membrane-bound peptides. It excludes some NMR models, low-resolution structures and theoretical models. The PDBTM database is created by scanning all PDB entries with the TMDET algorithm (25) and provides separate downloads for all helical transmembrane proteins. We search all three databases for new entries when updating *MP:PD*. *MP:PD* includes entries derived from various techniques such as electron crystallography, electron microscopy, solid-state NMR, solution NMR and X-ray diffraction. Theoretical models and peripheral membrane proteins are excluded. Internal cavities, internal waters, hydrogen bonds and internal packing densities are calculated for all entries (see ‘generation of database’), excluding those containing only backbone atoms or those resolved at low resolution (≥ 4.0 Å).

When applicable, the OPM database supplies quaternary complexes, i.e. biological units, provided by the authors or calculated by theoretical methods using PQS (26) or PISA (27). For PDB entries not listed in the OPM database, the first biological assembly was retrieved from the PDBe database (28) and sent to the PPM server, which calculates the transmembrane regions employing the same algorithm used for the entries in OPM (23). The transmembrane region is then defined by the membrane boundary planes given by OPM from insertion of quaternary complexes—rather than orientations of individual subunits or domains—into an implicit anisotropic solvent model of the lipid bilayer (29). Those residues having at least one atom lying within these planes were denoted as belonging to the transmembrane region. The calculation of biological units generally seems to be quite robust, but in some cases can lead to inaccurate definitions of the orientation of membrane protein structures relative to the membrane (24).

### Search functions

Entries and associated data of *MP:HD* can be accessed by PDB ID, PDB keywords, PDB title (22), OPM family / superfamily (23), MPstruc Subgroup and MPstruc Name searches. The search is generally case insensitive with white spaces separating query phrases. Query phrases can be concatenated by ‘+’ or ‘AND’ to perform combined searches, e.g. ‘rhodopsin + G-protein’, where both phrases must match. Quoted query phrases are also available to find phrases containing whitespaces, e.g. ‘M intermediate’ can be used to find entries related to bacteriorhodopsin's M intermediate state. Otherwise, results include all entries with a match in any of the query phrases, so that the query can be used to search for multiple PDB IDs ‘3dqb 3sn6 1c3w’.

By submitting the query, the user is forwarded to the results page listing all matched entries in a table. Single entries can be downloaded by mouse click or visualized by *Provi* (see next section). An info button provides information on PDB ID, PDB title, OPM family, OPM representative and OPM related entries, MPstruc subgroup and keywords. The results table can be sorted by clicking on the header of a column, i.e. PDB ID, experimental method, resolution, PDB title, packing density, water-, residue- and cavity count of the transmembrane spanning part, PDB keywords and various OPM (e.g. superfamily, family, species) or MPstruc (e.g. subgroup, name) related data. The sorting allows grouping of the data and facilitates selections. Rows can be selected using the mouse and standard keys: clicking on a row selects only that row. Holding the shift key does a range selection. Selection and deselection of individual rows can be achieved by holding the ‘ctrl’ key. The full table or the selected rows can be downloaded as a CSV file by clicking on the respective links just above the table. The zipped structure data are provided via a programatic database access.

### Visualization with *Provi*

*Provi* is a web browser-based visualization tool for protein structures and related data. It was built to allow immediate visualization of the analyzed structures. While relying heavily on *Jmol* (http://www.jmol.org) for 3D display its main features are the integrative display of structures along with associated datasets and a set of graphical user interface tools allowing focus on the most relevant structural aspects. Generally, the integrated display of low-energy waters alongside with membrane planes, internal packing density, hydrophobic cavities and hydrogen bonds helps to gain a more comprehensive view of the analyzed data and to derive structural aspects that would not be as evident when displayed separately. All experimentally determined internal water positions from the original PDB file can be shown alongside the newly assigned low-energy internal water positions.

## GENERATION OF DATABASE

### Internal cavities

Internal cavities are frequently found in protein domains with more than 150 amino acids (30). Internal cavities are defined here as internal packing defects large enough to enclose at least a spherical probe with 1.4 Å radius which approximates the Coulomb radius of a single water molecule. ‘Internal’ means that the cavity is largely buried within the protein interior. To differentiate buried from exposed protein atoms forming either internal or largely exposed cavities, we constructed a tight envelope around the protein by rolling a 2.8 Å sized spherical probe along the protein surface using the program MSMS (20). With this definition, we also include cavities that are partially accessible to water from the bulk phase, i.e. cavities placed at clefts of membrane proteins or within channels and pores restricted by narrow entranceways. However, we exclude wide open cavities and pockets lying at the protein surface that are the subject of substantially distinct computational approaches (31,32). The accuracy of determining solvent accessible surfaces e.g. of protein cavities can be improved if the radii of the cavity forming atoms are allowed to change depending on the polar or hydrophobic nature of the cavity (33). To calculate the shape of internal cavities, we are using a spherical probe of 1.4 Å, the Coulomb radius of water, to calculate the surface of polar cavities (i.e. cavities including internal water, for details see next paragraph) and a spherical probe of 1.7 Å, the van der Waals radius of water, to calculate the surface of hydrophobic cavities (i.e. cavities not containing water).

### Internal water and hydrogen bonds

Internal waters are defined as waters positioned no closer than 1.4 Å to the protein surface (see previous paragraph). To find internal water positions not listed in the PDB entry we conducted an exhaustive search of the program DOWSER (21). This program detects protein cavities and pockets and assesses their hydrophilicity in terms of energy interaction of a water molecule with the surrounding atoms. Water molecules with interaction energies <−10 kcal/mol are considered ‘low energy waters’ and are selected for output. After an initial run of the ‘dowserx’ script we applied various runs of the ‘dowser-repeat’ script until no additional low energy waters were detected. Because hetero atoms (e.g. ligands or ions) are not taken into account by DOWSER—no appropriate parameters were provided by that tool—therefore we did not place internal waters in contact distance to a heteroatom. As a result, all internal waters in close contact to hetero atoms contained in our database were those provided by the original PDB file. The positions of originally reported waters are refined by DOWSER, if low energy water can be placed at a given position. We decided to include the remaining 10% of experimentally determined internal waters in the final structure file, assuming that these waters were placed due to experimental constraints e.g. electron densities. Potential hydrogen bonds of internal water with cavity forming residues were identified with the HBexplore program, which selects all potential hydrogen bonds according to geometrical criteria (34).

### Packing densities

The atomic packing density quantifies the space between atoms. It allows a better approximation of van der Waals contacts and surfaces than a simple calculation of solvent excluded surfaces that does not respect packing defects enclosed therein. It uses two types of atomic volume, the van der Waals volume V(vdW) (inside the van der Waals radius), and the solvent excluded volume V(se) (a 1.4 Å layer cushioning the vdW sphere). The Voronoi Cell algorithm (19) calculates how much of the V(vdW) and V(se) is occupied by other atoms (see website for illustration). The packing density (PD) is then calculated from the remaining volumes V(vdW) and the sum of V(vdW) and V(se) using the formula PD = V(vdW)/[V(vdW) + V(se)]. The core algorithm to calculate atomic volumes is implemented in Delphi and an intermediate layer in Python. It calculates atomic volumes from PDB structures and produces modified PDB files from which packing densities and tabular reports containing average volumes and densities are calculated. We employed the widely used PROTOR radius set to define atomic volumes (35) which gives rise to slightly lower packing density values as when using the STOUTEN radii (36). As a result we obtained lower internal packing density values than in our previous analyses (3,5). The packing densities were calculated for the original PDB files without water and for our final structure files containing all newly assigned internal water.

### Technical notes

The webserver is based on the Flask framework (http://flask.pocoo.org/) and uses SQLite as its database. *Provi* relies on *Jmol* to display relevant aspects of protein structures. Its graphical user interface utilizes the jQuery JavaScript library augmented by a set of plugins to create the interface components and handle the interaction with the user.

## CONCLUSION AND FUTURE DIRECTION

Several computational biophysical tools were used to calculate internal packing densities, identify and characterize internal cavities and calculate their occupancy with internal water molecules for the alpha helical transmembrane proteins stored in *MP:PD*. For the transmembrane region, eight additional water positions per 100 residues were newly assigned on average. In this way, the number of internal waters is multiplied, compared with the original PDB files. Consistent with the strong negative correlation between structure resolution and water content observed for the original PDB file, the number of newly identified internal waters increases with decreasing structure resolution (see website for statistics). This correlation is abrogated by adding the newly assigned and refined waters to the original PDB file, indicating that the search for internal waters is largely exhaustive. A clear limitation of the present approach is that it does not assign new water positions contacting hetero atoms. These limitations, however, could be overcome in future by obtaining appropriate parameters for hetero atoms from other sources, allowing us to scan the close vicinity of hetero atoms for new water positions.

**Figure 1.**
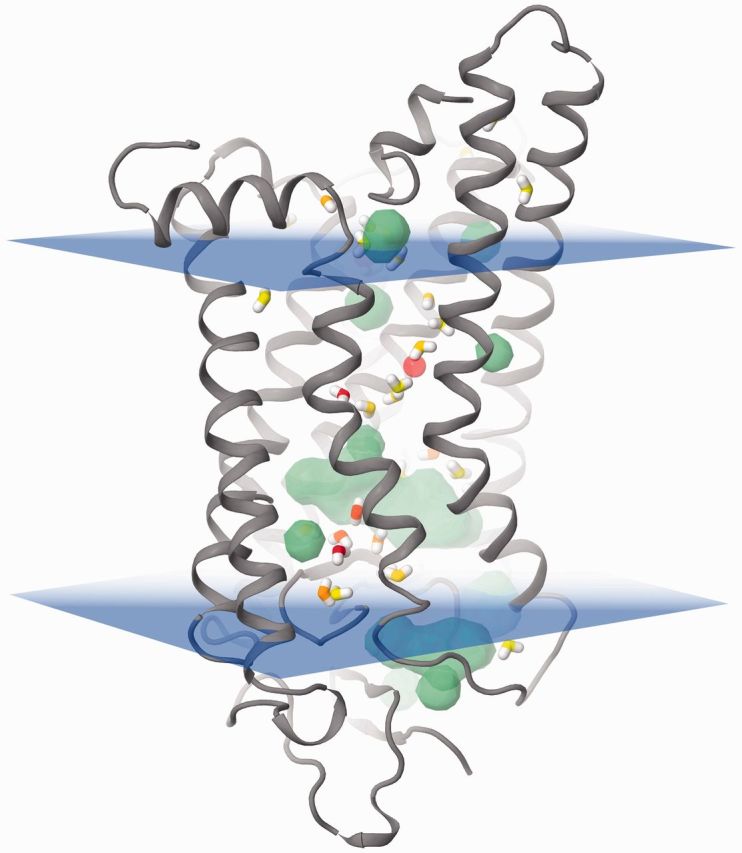
Refined water positions within the structure of the active GPCR opsin visualized with *Provi*. Low-energy waters in the active GPCR opsin with bound Gα C-terminal peptide (PDB-entry code: 3dqb) are shown as sticks colored from red (−30 kcal/mol) to yellow (−10 kcal/mol). A continuous wire of seven water molecules extends from the extracellular empty retinal binding pocket (in translucent green) located near the lower membrane plane (in translucent blue) up to the intracellular region of the receptor. This water wire includes only a single water determined by the original crystal structure analysis (depicted as translucent red ball). Six of these waters were also reported by crystal structure analysis of a structurally equivalent state of Meta II rhodopsin (PDB entry code: 2x72).

A functional role of internal waters for rhodopsin activation and function has been proposed lately by various approaches (10,13,14). A water-mediated hydrogen bonding network interconnecting the extracellular ligand binding pocket with the highly conserved D(E)RY motif at the cytosolic side was in fact identified by crystal structure analysis of Meta II rhodopsin (PDB entry code: 2x72) (14). As a result of the extensive search by DOWSER, additional waters are placed within this network and existing waters are repositioned such that a continuous water wire is emerging. The same water wire is observed in the *MP:PD* entry of opsin (PDB entry code: 3dqb), where six of the seven waters were newly assigned (Figure 1). This example indicates that the assignment of internal water used here is largely robust. It is therefore reasonable to assume that the additional water positions stored in *MP:DB* complement the structural information given by the original PDB files.

The transmembrane region of helical membrane proteins contains a reasonable number of hydrophobic cavities, i.e. internal cavities mainly built from nonpolar atoms that do not form energetically favourable interactions with internal waters. There is an ongoing controversial discussion on whether empty cavities in proteins exist or not (7). Hydrophobic cavities have been identified by NMR analysis using small gas molecules (6). Moreover, voids seem to play a dominant role in unfolding processes of proteins, as filling naturally occurring cavities stabilizes them against pressure denaturation (37). Hydrophobic cavities, however, are not necessarily packed with hydrophobic molecules, but may also contain water wires or clusters (7,38). Empty or partially empty cavities should also make helical membrane proteins more flexible allowing them to adopt various states or sub-states (3,9,39). Taken together, hydrophobic cavities seem to be important for the stability and function of proteins, but their specific role seems to depend on the substructural context. The integrative display of the *MP:PD* entries along with associated datasets helps to gain a more comprehensive view of the analyzed data and to derive structural aspects that would not be as evident when displayed separately.

## FUNDING

The Deutsche Forschungsgemeinschaft [DFG HI 1502/1-1, WO 1908/2-1, SFB 740]; European Research Council [ERC Advanced Grant TUDOR]. Funding for open access charge: The Deutsche Forschungsgemeinschaft [SFB 740/B6].

*Conflict of interest statement*. None declared.
